# Adequate Catchment Area Representation in Cancer Clinical Trials at NCI Designated Cancer Centers: The University of California Irvine Experience

**DOI:** 10.1002/cam4.71327

**Published:** 2025-11-04

**Authors:** Frank Lee, Aditya Mahadevan, Armon Azizi, Jennifer Valerin, Nataliya Mar, Deepa Jeyakumar, Farshid Dayyani

**Affiliations:** ^1^ University of California, Irvine School of Medicine Irvine California USA; ^2^ Department of Medicine University of California San Francisco San Francisco California USA; ^3^ Department of Medicine University of California San Diego San Diego California USA; ^4^ Division of Hematology‐Oncology, Department of Medicine, Chao Family Comprehensive Cancer Center, UC Irvine Health Orange California USA

## Abstract

**Background:**

Ensuring that clinical trial enrollment reflects the demographics and disease burden of a cancer center's catchment area is essential for improving trial accrual and the generalizability of research findings. We hypothesized that aligning the clinical trial portfolio with the needs of the catchment area can enhance enrollment and access to novel therapeutics.

**Methods:**

A retrospective cohort study was conducted at the University of California Irvine Chao Family Comprehensive Cancer Center (CFCCC), an National Cancer Institute‐designated cancer center serving Orange County (OC), California—the 6th largest populous county in the United States. Clinical trial enrollment data from 2015 to 2023 were analyzed using the CFCCC clinical research database. Patient demographics, tumor types, and trial enrollment patterns were compared with population‐level data from NIH SEER for both OC and the United States.

**Results:**

Between 2015 and 2023, 2317 participants were enrolled in clinical trials. Demographics were: White (66.4%), Asian (20.1%), Black (2.4%), American Indian/Alaska Native (0.7%), mixed/unknown (9.3%). Ethnicity: Non‐Hispanic (77.8%) vs. Hispanic (20.5%). Sex: Female (47.6%) vs. Male (52.3%). Age: < 70 years (73.3%) vs. ≥ 70 years (26.7%). Residence in low‐income/Health Professional Shortage Areas (HPSA): 44.8%. Trial phases included Phase I/II (35.6%), Phase II (25.4%), Phase II/III (2.8%), Phase III (26.6%), and Phase IV (0.3%). Study sponsors included Industry (61.1%), Institutional (23.6%), National (14.8%), and Externally Peer Reviewed (0.3%). Enrollment patterns reflected and, in some areas, exceeded the regional representation, particularly for Asian and Hispanic populations. Cancer‐type‐specific analysis showed higher enrollment for lung and liver cancers among Asians, breast cancer among Hispanics, and prostate cancer among Black patients.

**Conclusions:**

These findings suggest that the strategic alignment of the clinical trial portfolio with the cancer burden and demographics of the catchment area can enhance accrual. This catchment‐based approach offers a scalable model for improving clinical trial participation and ensuring the relevance of research to the communities served.

## 1 Introduction

Clinical trials play a critical role in advancing cancer care by offering early access to novel therapies and contributing to the development of future standards of care [1]. The external validity of these trials depends heavily on how well enrolled participants reflect the real‐world populations they are intended to serve [2]. However, trial populations may differ from broader populations based on various clinical and demographic characteristics, including gender, age, comorbidities, and geographic distribution [2]. Inadequate representation of the broader patient population in clinical trials may limit the applicability of research findings and the overall impact of new cancer treatments.

Ensuring that trial participants reflect the demographic and clinical composition of a cancer center's catchment area improves both enrollment and relevance of clinical research [3]. Although efforts have been made to improve representativeness in trial participation, gaps remain—particularly among certain demographic groups and regions—especially in randomized cancer clinical trials [4, 5, 6]. For example, previous studies report that individuals of Asian and Hispanic background comprise as little as 1% and 6%, respectively, of all trial enrollees [7]. These imbalances may impact the interpretation of results, especially when treatment responses vary by patient subgroup [3, 8].

Despite longstanding initiatives, including the NIH Revitalization Act and the ASCO‐ACCC Joint Research Statement, achieving proportional representation in clinical trials has proven difficult [5, 9]. Although reporting of participant demographics has improved, many trials—particularly those funded by industry—continue to omit such data [7, 10]. In fact, only 17% of participants in industry‐funded trials were reported as non‐White [11], highlighting persistent challenges in enrolling patients who reflect the broader U.S. population.

While numerous studies have assessed trial enrollment across specific cancers, few have analyzed clinical trial participation over an extended period at an National Cancer Institute (NCI)‐designated cancer center located in a minority‐majority county—a county in which more than 50% of the population identifies as a racial or ethnic minority [12]. Only 12.5% of NCI‐designated cancer centers are based in such counties, making them uniquely positioned to explore how trial enrollment can align with catchment area demographics [13].

To address this gap, we conducted a cross‐sectional observational study of patients enrolled in clinical trials at the University of California Irvine Chao Family Comprehensive Cancer Center (CFCCC), located in Orange County (OC), California. As a cancer center based in a minority‐majority region, CFCCC offers a valuable opportunity to assess how a portfolio tailored to the local disease burden and care access patterns may support broader clinical trial participation. We hypothesized that CFCCC would enroll patient populations at rates that reflect or exceed local demographic benchmarks, in part due to intentional portfolio design and regional accessibility.

## 2 Methods

Clinical trial enrollment data was collected from 2015 to 2023 from IRB‐approved interventional therapeutic cancer trials (as defined by the NCI) from the Chao Family Comprehensive Cancer Center (CFCCC) clinical trial management system (CTMS), Advarra OnCore [14]. OnCore is continuously updated and quality monitored by CFCCC's clinical operations team and reported to the NCI on a regular basis. Non‐therapeutic clinical trials were excluded from this study. Information about race/ethnicity (self‐reported per NCI guidelines), sex, study stage, income, cancer type, and study sponsor was collected for further analysis through an anonymized data pull. For this study, demographic groups were defined according to standard NCI classifications, with race categories including American Indian/Alaska Native, Asian, Black/African American, Native Hawaiian/Pacific Islander, and Latino/Hispanic identity for ethnicity [15]. Due to the exclusion of identifiable PHI, this study was deemed IRB‐exempt by the UC Irvine Institutional Review Board. Demographics were compared to data provided by the NIH SEER reports for both OC and the United States. The catchment area of CFCCC is defined as OC, California. For comparisons of discrete data, the chi‐squared statistical test was used. For comparisons of enrollment rates between different demographic groups and their general population, relative risk was calculated. The threshold for statistical significance was set at *p* = 0.05. All analyses were performed in STATA‐MP version 18.

## 3 Results

Between 2015 and 2023, 2315 subjects across three different UCI Health facilities were enrolled in clinical trials and included in the analysis. The cohort was 52% male (*n* = 1196). Most patients were White (1539 [66.4%]), Asian (467 [20.1%]), or Black (57 [2.4%]), and 477 (20.5%) were Hispanic. 1039 (44.8%) patients were from Low Income/Health Professional Shortage Areas (Table 1). The number of Hispanic patients under 60 years old was 307 (64.3%) as opposed to 685 (37.9%) for non‐Hispanics (Table S1).

**TABLE 1:** Sociodemographics of enrolled cancer trial patients at CFCCC between 2015 and 2023 (*N* = 2317).

Sociodemographic factors	Total: *N* (%)
Sex	
Male	1213 (52.3%)
Female	1104 (47.6%)
Age	
0–19	1 (0.04%)
20–29	43 (1.8%)
30–39	128 (5.5%)
40–49	296 (12.7%)
50–59	546 (23.5%)
60–69	680 (29.3%)
70–79	498 (21.4%)
80–89	116 (5.0%)
90+	8 (0.3%)
Unknown	1 (0.04%)
Race	
White	1539 (66.4%)
American Indian/Alaska Native	18 (0.7%)
Asian	467 (20.1%)
Black or African American	57 (2.4%)
Native Hawaiian/Pacific Islander	17 (0.7%)
Mixed Race	46 (1.9%)
Unknown	173 (7.4%)
Ethnicity	
Hispanic/Latino	477 (20.5%)
Non‐Hispanic/Latino	1804 (77.8%)
Unknown	36 (1.5%)
Income bracket	
Low income/HPSA	1039 (44.8%)
Non‐low income/HPSA	1278 (55.1%)
Trial information	
Study site	
UCI Health—Newport Beach	48 (2.0%)
UCI Health—Pacific Breast Care Center	26 (1.1%)
UCI Medical Center—Orange	2243 (96.8%)
Study phase	
I/II	826 (35.6%)
II	590 (25.4%)
II/III	65 (2.8%)
III	618 (26.6%)
IV	8 (0.3%)
N/A	210 (9.0%)
Top 10 cancer types	
Breast	275 (12.9%)
Lung and Bronchus	248 (11.6%)
Blood, Bone Marrow, & Hematopoietic System	184 (8.6%)
Prostate Gland	182 (8.5%)
Brain	165 (7.7%)
Bladder	142 (6.6%)
Liver	105 (4.9%)
Pancreas	91 (4.2%)
Ovary	90 (4.2%)
Skin	81 (3.8%)
Study sponsor	
Industry	1417 (61.1%)
Institutional	548 (23.6%)
National	345 (14.8%)
Externally peer‐reviewed	7 (0.3%)

**FIGURE 1:**
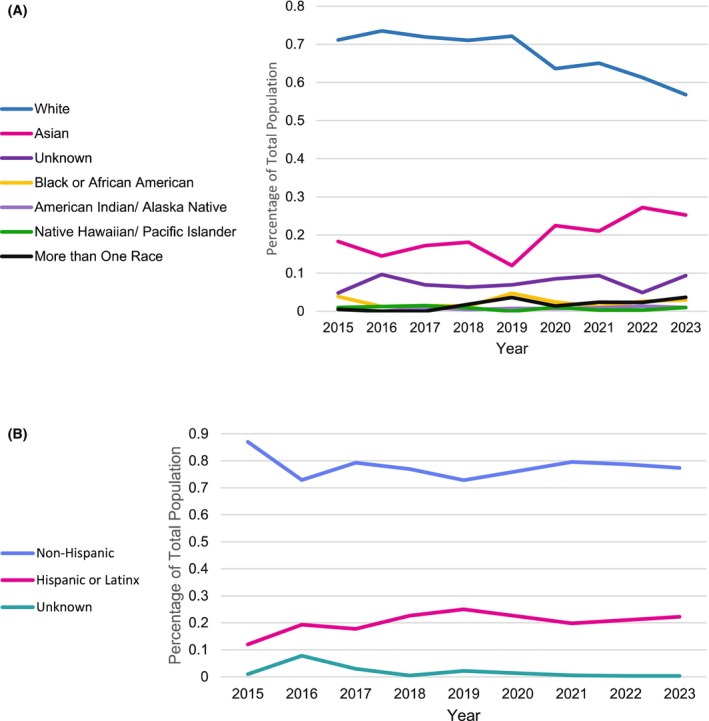
Trends in (A) race and (B) ethnicity at CFCCC trials from 2015 to 2023.

Enrollment increased overall from the years 2016 to 2021 (Figure 1). From 2021 onward, there were decreases in enrollment for White patients; however, Hispanic and Asian patients maintained their increased enrollment (Figure 1). Regarding relative enrollment rates, the rate of enrollment for Hispanic patients compared to the overall Hispanic patient population was higher than that of White patients (RR for Hispanic enrollment = 1.07, RR for White enrollment = 0.87). Additionally Asian patients were enrolled at higher rates compared to the general patient population as well (RR for Asian patient enrollment = 1.18).

Out of the 46 different cancer types at CFCCC, the 10 most common enrolled in order were Breast (275 [12.9%]), Lung (248 [11.6%]), Blood/Bone Marrow (184 [8.6%]), Prostate (182 [8.6%]), Brain (165 [7.7%]), Bladder (142 [6.6%]), Liver (105 [4.9%]), Pancreas (91 [4.2%]), Ovary (90 [4.2%]), and Skin (81 [3.8%]). When evaluating the study stages for patient enrollment, 826 (35.6%) of patients were enrolled in Phase I/II, 590 (25.4%) were enrolled in Phase II, 65 (2.8%) were enrolled in Phase II/III, 618 (26.6%) were enrolled in Phase III, and 8 (0.3%) were enrolled in Phase IV trials (Table 1).

Compared with national clinical trial enrollment, CFCCC enrolled a higher proportion of Asian patients (20.1% vs. 6.2%, Chi‐Sq *p* < 0.001) (Table 2). There was lower enrollment of Black (2.4% vs. 13.6%, Chi‐Sq *p* < 0.001) and White (66.4% vs. 75.4%, Chi‐Sq *p* < 0.001) patients when compared to national data. There was also a lower percentage enrollment of Hispanic patients compared to OC as a whole (20.5% vs. 34.0%, Chi‐Sq *p* < 0.001); however, this was comparable to Hispanic patient enrollment rates nationally (20.5% vs. 19.1%, N.S.).

**TABLE 2:** Comparison between CFCC, Orange County, and national demographics in 2023.

	CFCC	Orange County	National
Total	2317	3,150,372	333,287,557
Race			
White	1539 (66.4%)	2,176,907 (69.1%)	251,602,174 (75.4%)
American Indian/Alaska Native	18 (0.7%)	34,654 (1.1%)	4,382,234 (1.3%)
Asian	467 (20.1%)	734,036 (23.3%)	20,953,941 (6.2%)
Black or African American	57 (2.4%)	72,458 (2.3%)	45,399,743 (13.6%)
Native Hawaiian/Pacific Islander	17 (0.7%)	12,601 (0.4%)	878,808 (0.2%)
Mixed Race	46 (1.9%)	122,864 (3.9%)	10,070,657 (3.0%)
Unknown	173 (7.4%)	N/A	N/A
Ethnicity			
Hispanic/Latino	477 (20.5%)	1,071,126 (34.0%)	63,664,346 (19.1%)
Non‐Hispanic/Latino	1804 (77.8%)	2,079,246 (66.0%)	269,623,211 (80.8%)
Unknown	36 (1.5%)	N/A	N/A

**FIGURE 2:**
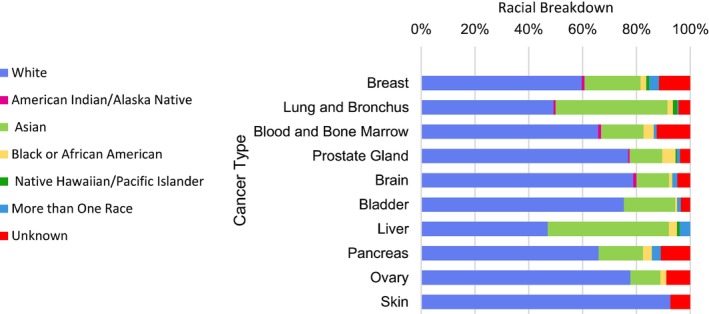
CFCCC demographics of clinical trial enrollment by cancer type from 2015 to 2023.

The clinical trials for certain cancer types disproportionately enrolled patients in specific race/ethnicity groups. Asian patients were enrolled at higher rates for clinical trials involving liver cancer (42.8% vs. 20.1% Asian enrollment overall, Chi‐Sq *p* < 0.001), lung and bronchus cancers (41.5% vs. 20.1%, Chi‐Sq *p* < 0.001), and similarly for breast (20.7% vs. 20.1%, N.S.) and bladder (19.0% vs. 20.1%, N.S.) (Table 3). There was 0% enrollment of Asian, American Indian, Black, Native Hawaiian, and Mixed Race patients in trials involving skin cancer (Table 3). Black patients were more likely to be enrolled in prostate cancer trials vs. Black enrollment overall (4.9% vs. 2.4%, Chi‐Sq *p* < 0.05) (Table 3). White patients were more likely to be enrolled in skin cancer trials vs. White enrollment overall (92.5% vs. 66.4%, Chi‐Sq *p* < 0.05) (Table 3). Hispanic patients were more likely to be enrolled in breast cancer trials vs. Hispanic enrollment overall (34.9% vs. 20.5%, Chi‐Sq *p* < 0.001) and less likely to be enrolled in lung and bronchus (9.6% vs. 20.5%, Chi‐Sq *p* < 0.001) and bladder cancer trials (8.4% vs. 20.5%, Chi‐Sq *p* < 0.001) (Table 4). The overall enrollment percentages for each race across the top ten cancer types are depicted in Figure 2.

**TABLE 3:** Cancer type vs. race.

Race	White	American Indian/Alaska Native	Asian	Black or African American	Native Hawaiian/Pacific Islander	Mixed Race	Unknown	Total
Cancer type								
Breast	164 (59.6%)	3 (1.0%)	57 (20.7%)	6 (2.1%)	3 (1.0%)	10 (3.6%)	32 (11.6%)	275
Lung and bronchus	122 (49.1%)	2 (0.8%)	103 (41.5%)	5 (2.0%)	4 (1.6%)	1 (0.4%)	11 (4.4%)	248
Blood and bone marrow	121 (65.7%)	2 (1.0%)	29 (15.7%)	7 (3.8%)	0 (0.0%)	2 (1.0%)	23 (12.5%)	184
Prostate gland	140 (76.9%)	1 (0.5%)	22 (12.0%)	9 (4.9%)	1 (0.5%)	2 (1.0%)	7 (3.8%)	182
Brain	130 (78.7%)	2 (1.2%)	20 (12.1%)	2 (1.2%)	0 (0.0%)	3 (1.8%)	8 (4.8%)	165
Bladder	107 (75.3%)	0 (0.0%)	27 (19.0%)	1 (0.7%)	0 (0.0%)	2 (1.4%)	5 (3.5%)	142
Liver	47 (44.7%)	0 (0.0%)	45 (42.8%)	3 (2.8%)	1 (0.9%)	4 (3.8%)	5 (4.7%)	105
Pancreas	60 (65.9%)	0 (0.0%)	15 (16.4%)	3 (3.2%)	0 (0.0%)	3 (3.2%)	10 (10.9%)	91
Ovary	70 (77.7%)	0 (0.0%)	10 (11.1%)	2 (2.2%)	0 (0.0%)	0 (0.0%)	8 (8.8%)	90
Skin	75 (92.5%)	0 (0.0%)	0 (0.0%)	0 (0.0%)	0 (0.0%)	0 (0.0%)	6 (7.4%)	81
Total	1539 (66.4%)	18 (0.7%)	467 (20.1%)	57 (2.4%)	17 (0.7%)	46 (1.9%)	173 (7.4%)	2317

**TABLE 4:** Cancer type vs. ethnicity.

Ethnicity	Hispanic	Non‐Hispanic	Unknown	Total
Cancer type				
Breast	93 (34.9%)	173 (65.0%)	0 (0.0%)	266
Lung and bronchus	24 (9.6%)	222 (89.5%)	2 (0.8%)	248
Blood and bone marrow	43 (23.3%)	139 (75.5%)	2 (1.0%)	184
Prostate gland	18 (9.8%)	164 (90.1%)	0 (0.0%)	182
Brain	37 (22.4%)	126 (76.3%)	2 (1.2%)	165
Bladder	12 (8.4%)	130 (91.5%)	0 (0.0%)	142
Liver	25 (23.8%)	80 (76.1%)	0 (0.0%)	105
Pancreas	23 (25.2%)	68 (74.7%)	0 (0.0%)	91
Ovary	16 (17.7%)	70 (77.7%)	4 (4.4%)	90
Skin	9 (11.1%)	70 (86.4%)	2 (2.4%)	81
Total	477 (20.5%)	1804 (77.8%)	36 (1.5%)	2317

When evaluating enrollment rates by trial phase, we found that Asians were enrolled at higher rates in Phase I/II trials vs. Asian enrollment overall (24.4% vs. 20.1%, Chi‐Sq *p* < 0.01) (Table 5). Additionally, Hispanic patients were enrolled at higher rates in phase II trials vs. Hispanic enrollment overall (25.4% vs. 20.5%, Chi‐Sq *p* < 0.01) (Table 6). Race and ethnicity were otherwise not associated with the phase of trial enrollment in our data.

**TABLE 5:** Race vs. trial phase.

Race	White	American Indian/Alaska Native	Asian	Black or African American	Native Hawaiian/Pacific Islander	Mixed Race	Unknown	Total
Trial phase								
Phase I/II	534 (64.6%)	6 (0.7%)	202 (24.4%)	19 (2.3%)	5 (0.6%)	18 (2.1%)	42 (5.0%)	826
Phase II	363 (61.5%)	5 (0.8%)	130 (22.0%)	12 (2.0%)	5 (0.8%)	16 (2.7%)	59 (10.0%)	590
Phase II/III	48 (73.8%)	0 (0.0%)	11 (16.9%)	2 (3.0%)	1 (1.5%)	0 (0.0%)	3 (4.6%)	65
Phase III	413 (66.8%)	4 (0.6%)	107 (17.3%)	21 (3.3%)	5 (0.8%)	12 (1.9%)	56 (9.0%)	618
Phase IV	3 (37.5%)	0 (0.0%)	3 (37.5%)	1 (12.5%)	1 (12.5%)	0 (0.0%)	0 (0.0%)	8
N/A	178 (84.7%)	3 (1.4%)	14 (6.6%)	2 (0.9%)	0 (0.0%)	0 (0.0%)	13 (6.1%)	210
Total	1539 (66.4%)	18 (0.7%)	467 (20.1%)	57 (2.4%)	17 (0.7%)	46 (1.9%)	173 (7.4%)	2317

**TABLE 6:** Ethnicity vs. trial phase.

Ethnicity	Hispanic	Non‐Hispanic	Unknown	Total
Trial phase				
Phase I/II	164 (19.8%)	656 (79.4%)	6 (0.7%)	826
Phase II	150 (25.4%)	434 (73.5%)	6 (1.0%)	590
Phase II/III	10 (15.3%)	52 (80.0%)	3 (4.6%)	65
Phase III	138 (22.3%)	470 (76.0%)	10 (1.6%)	618
Phase IV	0 (0.0%)	8 (100%)	0 (0.0%)	8
N/A	15 (7.1%)	184 (87.6%)	11 (5.2%)	210
Total	477 (20.5%)	1804 (77.8%)	36 (1.5%)	2317

When evaluating enrollment rates by trial sponsor type, we found that Asian patients were more likely to be enrolled in Industry‐sponsored trials vs. Asian enrollment overall (22.0% vs. 20.1%, Chi‐Sq *p* < 0.05), and that Black patients were less likely to be enrolled in institutional trials vs. Black enrollment overall (1% vs. 2.4%, Chi‐Sq *p* < 0.05) (Table 7). Hispanic patients were less likely to be enrolled in Industry‐sponsored trials (18.8% vs. 20.5% enrollment overall, Chi‐Sq *p* < 0.05) (Table 8).

**TABLE 7:** Race vs. sponsor type.

Race	White	American Indian/Alaska Native	Asian	Black or African American	Native Hawaiian/Pacific Islander	Mixed Race	Unknown	Total
Sponsor type								
Industry	911 (64.2%)	9 (0.6%)	314 (22.0%)	38 (2.6%)	10 (0.7%)	29 (2.0%)	106 (7.4%)	1417
Institutional	387 (70.6%)	6 (1.0%)	95 (17.3%)	6 (1.0%)	3 (0.5%)	7 (1.2%)	44 (8.0%)	548
National	239 (69.2%)	3 (0.8%)	55 (15.9%)	13 (3.7%)	4 (1.1%)	8 (2.3%)	23 (6.6%)	345
Externally peer reviewed	2 (28.5%)	0 (0.0%)	3 (42.8%)	0 (0.0%)	0 (0.0%)	2 (28.5%)	0 (0.0%)	7
Total	1539 (66.4%)	18 (0.7%)	467 (20.1%)	57 (2.4%)	17 (0.7%)	46 (1.9%)	173 (7.4%)	2317

**TABLE 8:** Ethnicity vs. sponsor type.

Ethnicity	Hispanic	Non‐Hispanic	Unknown	Total
Trial phase				
Industry	267 (18.8%)	1131 (79.8%)	19 (1.3%)	1417
Institutional	123 (22.4%)	411 (75.0%)	14 (2.5%)	548
National	87 (25.2%)	255 (73.9%)	3 (0.8%)	345
Externally peer reviewed	0 (0.0%)	7 (100%)	0 (0.0%)	7
Total	477 (20.5%)	1804 (77.8%)	36 (1.5%)	2317

## 4 Discussion

### 4.1 Summary of Key Results

While there are 72 NCI‐designated cancer centers in the United States, only nine are located in minority‐majority counties [13]. To our knowledge, this is the first cross‐sectional study to present longitudinal patient data from an NCI center located in a minority‐majority county. Overall, we have found robust relative enrollment for Asian and Hispanic populations, and representative enrollment for Black populations. We have also found specific cancer trials that enrolled a disproportionate number of minorities, and certain trends in minority enrollment over time. Lastly, we found a high enrollment rate for individuals designated as Low Income/Health Professional Shortage Areas (LI/HPSA).

### 4.2 Asian Recruitment

Overall, recruitment of minorities at our center was robust. Compared to national census data, our cohort showed significantly higher recruitment of Asian populations [16]. Robust Asian‐American trial enrollment at CFCCC can be attributed to many factors, including a tailored clinical trial portfolio addressing high‐incidence malignancies in this population (Table S2). To this end, OC is home to the largest population of Vietnamese individuals outside of Vietnam, a population with relatively high rates of hepatitis B/C infection and increased risk for hepatocellular carcinoma (HCC) [17]. Asian‐Americans also experience a higher burden of stomach and nasopharyngeal cancers, well‐represented in the CFCCC trial portfolio [18, 19]. Despite historic under‐enrollment of Asians in cancer clinical trials to date [20, 21], these study findings highlight the feasibility of Asian‐American clinical trial enrollment.

### 4.3 Hispanic Recruitment

While our recruitment of Hispanic individuals still lags behind the proportion of Hispanics living in OC [19], we found that our center enrolled a higher proportion of Hispanic patients than expected. This contrasts with previous studies suggesting that Hispanic patients have been historically underenrolled in cancer trials, with enrollment numbers often trailing in the single digits [22, 23]. Additionally, Hispanic patients are known to have lower rates of insurance coverage [24]. As a result, UCI's status as a safety‐net hospital—defined by the NIH as a provider of significant healthcare services to individuals with no insurance or Medicaid—may have facilitated increased trial enrollment by enabling these individuals to participate in clinical trials regardless of their ability to pay [25]. While further research should be conducted to understand and address regional barriers to clinical trial participation, our data suggest that tailoring trials to address the healthcare needs of Hispanic patients could result in stronger enrollment rates despite these challenges.

### 4.4 Black Recruitment

The proportion of Black individuals at CFCCC closely matches the Black population in OC. While Black patients represent a small portion of the OC population, their representation in clinical trials remains important. However, given the smaller proportion of Black individuals in the local population, it is challenging to draw definitive conclusions regarding Black clinical trial enrollment at CFCCC. Historically, Black populations in the United States have been underrepresented in cancer clinical trials [26], which is concerning due to the persistently low survival rates across many cancer types in this group [27]. The underenrollment of Black patients in trials is likely influenced by several factors, including a lack of trials that match a patient's cancer type, restrictive eligibility criteria, and insurance limitations [28, 29]. Additionally, historical events like the Tuskegee Syphilis Study have contributed to medical mistrust within Black communities, further hindering trial enrollment. A recent study at an NCI cancer center found significantly higher enrollment of Black patients; however, this likely reflects differences in population composition between the cancer center's referral base, rather than significant differences in institutional strategies [30]. Our results represent a positive step toward achieving greater alignment between clinical trial participation and the local population's demographics for Black individuals in cancer research.

### 4.5 Enrollment Rates Over Time

We found that since 2015, White enrollment steadily decreased while Asian enrollment steadily increased. There was a decrease in Asian enrollment between 2020 and 2021, potentially owing to the impact of the COVID‐19 pandemic [31]. While Hispanic enrollment at CFCCC steadily increased from 2015 to 2019, consistent with a study aggregating NCI‐designated trials, it has remained largely unchanged from 2019 onwards [32]. While cancer clinical trial enrollment among minority populations in OC has remained relatively stagnant in recent years, these trends are not consistent with national data indicative of a widening diversity gap in cancer clinical trial enrollment during the COVID‐19 pandemic [33]. Additional research is needed to further characterize these trends.

### 4.6 Cancer‐Specific Enrollment Rates

Clinical trial enrollment by race varied significantly by cancer type in our cohort. In our cohort, Asian Americans were enrolled at significantly higher rates in liver and lung cancer clinical trials compared to non‐Hispanic Whites, reflective of the high incidence of hepatocellular and lung carcinoma among Asian‐Americans [18, 19]. Of note, Hispanic patients enrolled at higher rates than non‐Hispanic Whites for breast cancer, despite experiencing significantly lower rates of breast cancer compared to non‐Hispanic Whites [34]. Black men are disproportionately affected by prostate cancer, and Black individuals participated in prostate cancer trials at CFCCC at significantly higher rates than expected [35]. Of note, non‐white patients enrolled at significantly lower rates in skin cancer clinical trials, which can be partially attributed to lower incidence of skin cancer in these populations [36]. Overall, minority participation in tumor‐specific trials at CFCCC was in line with their incidence in these respective populations, representing a significant step forward in cancer clinical trial representation compared to prior national trends [34, 37].

### 4.7 Trial Phase

Race‐based differences in trial enrollment extended to trial phase as well with Asian Americans and Hispanics being more likely to enroll in early phase trials. Historically, strict eligibility criteria and other barriers have prevented minorities from participating in earlier studies, and many benefits can be derived from having more diverse early phase trials [38]. Asians are known to have higher Phase I enrollment rates in the literature, and our results corroborate this finding [22]. Conversely, Hispanics are historically underrepresented in Phase II/Phase III trials [39]; our distinct findings indicate the possibility of growing Hispanic population recruitment in early phase trials (like Phase I and II). Minority recruitment in early phase trials has several benefits, namely providing valuable safety data on the side effect profiles of novel chemotherapeutics in minority populations. This is of particular importance, given that ethnic variation in allelic expression may lead to differences in pharmacokinetics and subsequently tolerability among various minority groups [40, 41]. Our results highlight the feasibility of recruiting historically underenrolled minorities in early phase clinical trials, representing a positive step toward equity in cancer care.

### 4.8 Participation by Income

Lastly, a significant proportion of our trial participants (44.8%) were designated as LI/HPSA individuals, defined as a population or geographic area lacking healthcare professionals in primary care, health care, and dental care [42]. Prior literature suggests that low‐income individuals are less likely to participate in cancer clinical trials due to financial strain or be offered the opportunity to enroll in clinical trials [43, 44, 45]. In stark contrast, the high percentage of individuals from these areas suggests that it is possible to achieve equitable rates of enrollment regardless of income level. This may be partially explained by UC Irvine's designation as a safety net hospital, which may have facilitated enrollment for low‐income individuals without fear of financial expenditures.

### 4.9 Practical Implications and Strategies

The practical implications of this study stem from the strategies used to incorporate the needs of the local catchment area into an accessible, tailored trial portfolio aimed at meeting the unmet needs of the catchment population.

**FIGURE 3:**
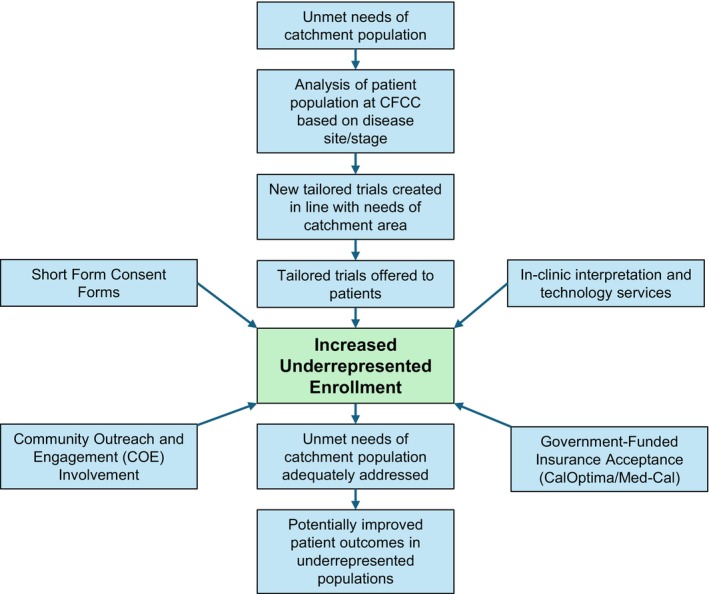
CFCCC strategies to address unmet needs in the catchment population.

At CFCCC, annual reviews of patient demographic data, along with cancer prevalence and stage statistics, are conducted. Disease‐oriented teams integrate this data into meetings designed to assess each therapeutic trial's potential for accrual, prioritizing those that address gaps in cancer care within the local population. This allows for a data‐driven approach to trial selection, targeting the unmet needs of our catchment area (Figure 3). For example, due to the high incidence of gastric cancer and HCC in our region, efforts are made to enroll patients in trials specifically targeting these malignancies. This strategy aligns with one of the core mandates of NCI‐designated cancer centers: to address the unique healthcare needs of the communities they serve. By prioritizing clinical trials that reflect the local disease burden rather than focusing solely on scientific novelty, we can more effectively meet the needs of our community, especially for Asian and Hispanic populations.

In addition to trial selection, ensuring accessibility for underinsured populations was key to achieving representative enrollment. Accepting public insurance programs, such as CalOptima and Medi‐Cal, has reduced financial barriers and expanded trial participation among low‐income patients, as reflected in nearly half of our patients being designated as from LI/HPSA. Furthermore, the integration of the Office of Community Outreach and Engagement (COE) from the early stages of trial design ensures that study protocols align with the linguistic, cultural, and logistical needs of the catchment area. For example, simplified informed consent forms, available in multiple languages, have enhanced comprehension and participation for non‐English‐speaking patients. Additionally, in‐clinic resources such as iPads with translation services and in‐person interpreters for commonly spoken languages, including Spanish and Vietnamese, have facilitated patient‐provider communication.

On a broader scale, participation in data‐sharing initiatives with other NCI‐designated cancer centers has fostered the exchange of best practices, further refining strategies for improving enrollment in clinical trials [46]. These concerted efforts contribute to a more inclusive research environment, ultimately enhancing the relevance and availability of novel cancer treatments for various populations.

### 4.10 Limitations

This study has some limitations. As a single‐center study, our findings may not be generalizable outside of the CFCCC catchment area. Additionally, demographic data on factors such as gender identity, sexual orientation, and primary language were not collected, which could provide further insight into trial enrollment trends among our patient population. Furthermore, because this registry data was directly extracted from the CFCCC database, we do not have access to specific information on patient income, insurance coverage, distance traveled, and other granular data. These variables, as well as clinical variables including comorbidities and performance status may influence the likelihood of trial enrollment. Investigator‐level differences may play a role in trial offering, though this is difficult to quantify. Lastly, as a quantitative study, we were unable to capture qualitative patient data regarding incentives and barriers to clinical trial enrollment.

## 5 Conclusion

Despite the presence of numerous barriers to clinical trial enrollment, our findings suggest that populations served at CFCCC are equally or more likely to enroll in clinical trials compared to White participants, when the trial portfolio and patient access are tailored to the needs of the local population. Our results, particularly among Asian and Hispanic populations, demonstrate that robust enrollment in clinical trials is feasible at an NCI cancer center when a tailored approach considers the local population in mind. Researchers should also be encouraged to consider the catchment area when designing investigator‐initiated trials. Additionally, strong cancer registry support is essential to compile timely and accurate data on the demographics and diagnoses seen at each cancer center, with updates ideally occurring on a regular basis (e.g., annually) to inform clinical trial portfolio management. Overall, further research is needed to investigate factors that influence clinical trial participation, with a focus on promoting greater representation and ensuring adequate cancer care.

## Author Contributions

**Frank Lee:** writing – original draft, writing – review and editing, conceptualization, methodology, software. **Aditya Mahadevan:** supervision, writing – original draft, writing – review and editing, conceptualization, methodology. **Armon Azizi:** software, formal analysis, project administration, methodology, validation, visualization. **Jennifer Valerin:** supervision, writing – review and editing. **Nataliya Mar:** data curation. **Deepa Jeyakumar:** data curation. **Farshid Dayyani:** conceptualization, investigation, supervision, writing – review and editing, data curation, resources.

## Ethics Statement

The UC Irvine Institutional Review Board Ethics Committee approved this study.

## Conflicts of Interest

Dr. Dayyani has served on advisory boards, speaker bureaus, or consulted with Astellas, Astrazeneca, Eisai, Exelixis, Ipsen, Jazz, Servier, Sirtex, and Takeda. Dr. Valerin has served on the speaker bureau and consulted with Astrazeneca and Tempus, respectively. Other authors declare no conflicts of interest.

## Supporting information


**Table S1:** Under over 60 versus ethnicity.

**Table S2:** Trial type by year.

## Data Availability

In accordance with the FAIR principles required for data access, the authors confirm that the data supporting these findings will be made publicly available through direct data pulled from the Chao Family Comprehensive Cancer Center (CFCCC) clinical trial management system (CTMS), Advarra OnCore via an appropriate text format.
